# The power to help or harm: student perceptions of transgender health education using a qualitative approach

**DOI:** 10.1186/s12909-023-04761-9

**Published:** 2023-11-07

**Authors:** Whitney Linsenmeyer, Katie Heiden-Rootes, Theresa Drallmeier, Rabia Rahman, Emily Buxbaum, Katherine Walcott, Willow Rosen, Beth (Eli) Gombos

**Affiliations:** 1 Department of Nutrition and Dietetics, Saint Louis University, 3437 Caroline Street, Room 3076, St. Louis, MO 63105 USA; 2 Department of Family and Community Medicine, Saint Louis University School of Medicine, 1008 S. Spring Avenue, 3rd Floor, St. Louis, MO 63110 USA; 3 Department of Speech, Language & Hearing Sciences, Saint Louis University, 3750 Lindell Blvd., Suite 23, St. Louis, MO 63108 USA; 4 Willow Rosen Consulting, St. Louis, 63116 USA; 5 Independent Consultant, St. Louis, MO 63118 USA

## Abstract

**Background:**

Lack of transgender health education among health professional education programs is a limitation to providing gender-affirming care. Educational interventions have advanced in the past decade using a variety of pedagogical approaches. Although evidence supports that educational interventions can significantly improve student knowledge, comfort levels, preparedness, and clinical skills, few studies have addressed student perceptions of or receptiveness towards transgender health education. The study purpose was to explore student perceptions of transgender health education using a qualitative approach.

**Methods:**

We utilized a basic qualitative design to explore student perceptions of transgender health education at a Catholic, Jesuit institution. Participants were medical students (n = 182), medical family therapy students (n = 8), speech, language and hearing sciences students (n = 44), and dietetic interns (n = 30) who participated in an Interprofessional Transgender Health Education Day (ITHED) in partnership with transgender educators and activists. Participants completed an online discussion assignment using eight discussion prompts specific to the ITHED sessions. Data were analyzed using the constant comparative method and triangulated across four medical and allied health programs.

**Results:**

A total of 263 participants provided 362 responses across eight discussion prompts. Three major themes resulted: (1) The Power to Help or Harm, (2) The Responsibility to Provide Health *Care*, and (3) A Posture of Humility: Listen and Learn. Each theme was supported by three to four subthemes.

**Conclusions:**

Health professional students were highly receptive towards transgender health education delivered by transgender community members. First-person accounts from session facilitators of both positive and negative experiences in healthcare were particularly effective at illustrating the power of providers to help or harm transgender patients. Reflection and constructive dialogue offers students an opportunity to better understand the lived experiences of transgender patients and explore their identities as healthcare providers at the intersection of their religious and cultural beliefs.

**Supplementary Information:**

The online version contains supplementary material available at 10.1186/s12909-023-04761-9.

## Background

The transgender population is poorly served by the United States healthcare system. One in three transgender adults who saw a healthcare provider in 2015 reported at least one negative experience such as being refused treatment, verbal harassment, physical or sexual assault, or having to teach the provider about their identity [1]. As a result, physical and mental health disparities have persisted in this population including severe psychological distress, suicidality, and HIV [1, 2].

Lack of transgender health education among health professional education programs is a limitation to providing gender-affirming care. Most medical students receive little or no training on transgender health [3, 4]. Educational interventions have advanced in the past decade for medical students and residents [4–26] as well as a multitude of other healthcare learners [27–35]. A variety of pedagogical approaches have been described including lectures, videos, case studies, online modules, simulations, clinical rotations, small group discussions, panel discussions, live patient interviews, and reflective writing assignments [4]. Some programs have sought to integrate transgender health-related content throughout medical or nursing curricula; [21, 24, 35] others have created an elective course or certificate program [22, 30].

Evidence supports that educational interventions can significantly improve student knowledge, comfort levels, preparedness and clinical skills [5–35]. However, few studies have addressed student perceptions of or receptiveness towards transgender health education. Attention to student perceptions is warranted given the highly politicized and often divisive nature of transgender health-related issues, such as the criminalization of medical providers for providing medical interventions for adolescents [36]. A record 70 anti-lesbian, gay, bisexual, transgender and queer (LGBTQ) laws were enacted in 2023, including 15 laws banning gender-affirming care for transgender youth and seven laws regarding the permissibility of misgendering transgender students [37]. In addition to state-level laws, religiously affiliated institutions may face restrictions on gender-affirming care, such as the recent move towards banning gender-affirming care at Catholic hospitals [38].

Student perceptions of transgender health will reasonably influence how they receive training on a politicized and controversial topic. A small number of studies using pre-test, post-test survey designs reported that educational interventions improved student attitudes, or what they think about transgender patients [7, 10, 15, 25, 27, 35]. Student attitudes were measured by their understanding of issues faced by transgender patients, [7] the appropriateness of gender-affirming medicine, [10] or their agreement with statements such as, “Identifying as a transgender should be considered a psychiatric illness” [25] or “As a nurse, I feel it is important for me to know about my patient’s gender identity.” [35]. Given the complex layers of state-level bans, restrictions from religiously affiliated institutions, and personal beliefs, further research is needed to examine student in-depth perceptions across health professions and at institutions that vary by location, religious affiliation, mission, and student demographics, among other characteristics.

Lastly, insight into student perceptions of transgender health education may inform clinical educators seeking to incorporate this content into their curricula. Clinical educators may be weighing the landscape of anti-LGBTQ laws in their state, the climate of their home institution, and the scope of practice regarding transgender healthcare in their discipline. Rich and detailed analyses may reveal nuances of student perceptions on the topic in order to inform their pedagogical approach.

### Study purpose and aims

The study purpose was to explore student perceptions of transgender health education at a Catholic, Jesuit institution. The aims were to (1) describe students’ perceptions of the importance of education specific to transgender health within their training, (2) characterize students’ beliefs about their professional identities as future healthcare providers who would one day be providing care for transgender patients, and (3) capture students’ reflections of the broader educational, political and social systems that impact the health of the transgender community. The Institutional Review Board of the authors’ home institution approved this study.

## Methods

### The interprofessional transgender health education day

The ITHED was a one-day, in-person training consisting of lectures, panel discussions, a guided reflection, and breakout sessions. Transgender educators provided lectures including Trans 101: Introduction to Transgender Health, Trans 102: Introduction to Gender-Affirming Medical Care and Transitioning, and the Panel Discussion with Transgender Community Members. Students attended two breakout sessions of their choosing related to topical issues in transgender health, clinical skills building, or small group discussions with faith leaders. Supplement 1 details the itinerary, learning objectives and breakout session descriptions.

### Study design and theoretical framework

We utilized a qualitative approach to explore student perceptions of transgender health education using a basic qualitative design, which is appropriate when the study purpose is to understand how participants interpret their experiences, construct their worlds, and make meaning from their experiences [39]. Our theoretical framework was informed by critical pedagogy, where the goal is to critique, challenge, and transform existing conditions, and teaching itself is viewed as a political act [40]. A critical pedagogical lens was necessitated by the pervasiveness of gender-based stigma and discrimination within healthcare, education, and broader society, as well as the politicized nature of transgender healthcare [36, 40]. Critical pedagogy informed the intervention in that the majority of the content centered on the history of transgender people, the current state of transgender healthcare access, and the experiences of transgender patients in the healthcare system—rather than specifics of gender-affirming medicine such as prescribing hormone therapy. This was reflected in the question prompts that required students to comment on the broader sociocultural environment (Table 1), i.e. *How have societal messages about gender evolved over the past several decades?* Our theoretical framework was further informed by the Jesuit value of Cura Personalis, or preparation of our students to provide routine and respectful care that that honors the dignity of the whole person [41]. This value informed the intervention as a whole in that the research team trains students to provide routine and respectful care for all patients, which has become especially critical for transgender patients where healthcare access may be threatened [37]. This value also informed the process of data analysis where the research team sought to understand how participants viewed themselves as future healthcare providers who would one day be providing care for transgender patients (aim 2), which may align or conflict with their personal views on gender diversity. Lastly, we relied on the Jesuit practice of reflection in professional identity development, which informed the data collection methods [42].

**Table 1:** Online Discussion Prompts Following the ITHED Participation

ITHED Session	Discussion Prompt	Number of Responses by Program				
		Medicine	Medical Family Therapy	Speech, Language & Hearing Sciences	Nutrition & Dietetics	Total
Panel of Transgender Community Members	What was most surprising or revealing to you about the experiences of the members of the transgender panel? How might your increased knowledge of transgender healthcare influence your future clinical practice?	45	4	12	7	68
Follow-up Discussion with Transgender Panel Members	What was most impactful about hearing directly from the transgender community?	22	1	7	0	30
Sex Under the Gender Expansive Umbrella	What useful clinical pearls can you share that will help a clinician obtain a routine sexual history of an LGBTQ patient?	21	3	0	0	24
Gender-affirming Communication: A Skills Workshop	How does language help or harm transgender patients and clients?	25	0	19	4	48
TransParent-ing: A Panel Discussion with Families of Transgender Youth	What did you learn about the needs of parents of transgender youth in healthcare?	51	3	21	31	106
Loitering with Intent: A Conversation with Nancy Corcoran, CSJ	What are ways that faith communities can benefit the health outcomes of transgender patients?	20	0	9	7	36
PrEP and HIV in the Gender Diverse Population	What are the critical takeaways for healthcare providers to know about PrEP and HIV in the gender diverse population?	26	1	0	8	35
“But What About…” Safe Space to Ask Lingering Questions	How have societal messages about gender evolved over the past several decades?	14	0	1	0	15
Total						362

### Participants and setting

Participants were graduate students in health professional education programs at a Midwestern, Catholic, Jesuit university with an affiliated medical school. Medical students (n = 182), family medical therapy students (n = 8), speech, language and hearing sciences students (n = 44), and dietetic interns (n = 30) were required to attend the Interprofessional Transgender Health Education Day (ITHED). Others attended but were not included in the research portion due to logistic reasons, including physician assistant students, occupational therapy students, and staff of the university’s student health center.

### Data collection and instrumentation

Following ITHED attendance, students participated in an online reflection and discussion assignment in their respective programs. Students were required to respond to at least one discussion prompt and comment on at least one post of their peers within seven days following the ITHED. The eight discussion prompts pertained to the lectures, panel, and breakout sessions, and are detailed in Table 1. The assignment was required, but permitting the research team to utilize their responses as data for research purposes was optional; those wishing to opt out of the research informed the primary investigator. In order to minimize pressure that students may have felt to participate in the research, the primary investigator was not the instructor or program director of any of the students who participated in the ITHED. Data were de-identified, aggregated, and organized in Excel; one sheet was designated for each discussion prompt and each comment was labeled by discipline type.

### Data analysis and strategies of trustworthiness

Data were analyzed inductively using the constant comparative method [39]. Two researchers read the responses pertaining to each question prompt and open coded the data. The first two researchers then used axial coding across all of the question prompts to relate the codes to one another and begin grouping them. A third researcher reviewed the codes and provided feedback. Selective coding was used to begin refining the codes and groupings. The research team then constructed the codes into themes and subthemes and reported them by frequency. All members of the research team reviewed the themes, subthemes, codes, and raw data, provided feedback, and formed a consensus to ensure the findings were an authentic representation of the data. Participant quotes to support the findings were reported verbatim; pseudonyms were used to protect confidentiality. Strategies of trustworthiness included two forms of triangulation: collecting data from four medical and allied health programs, and the involvement of three researchers in data analysis. The study methodology was reported in detail to support transparency. Member checking was not attempted given the large number of study participants [1]. Lastly, the authors utilized the American Psychological Association Journal Article Reporting Standards (JARS) to ensure all required elements were reported [43].

### Research team

The research team was comprised of clinician scientists and community educators. The clinician scientists are interdisciplinary in nature (family medicine, medical family therapy, speech language pathology, nutrition and dietetics), hold academic appointments in the medical school or college of allied health, and are experienced in gender-affirming healthcare and research within our respective disciplines. Two researchers are trained in qualitative methods. The community educators led the ITHED delivery and have worked with community-based organizations that serve the transgender community. Their community work spans pragmatic social support such as assisting transgender community members with housing, job applications, medical care referrals, name change processes and community engagement. These community educators also have experience working with other healthcare providers on workplace culture and change, provider training and business growth related to transgender health.

### Ethical considerations

We aimed to honor the guidelines and ethical considerations for transgender health research posed by Adams and colleagues [44] by closely collaborating with transgender educators in delivering the ITHED, ensuring language in the study materials and manuscript was non-stigmatizing, and following IRB protocols for informed consent and participant confidentiality.

## Results and discussion

A total of 264 students participated in the ITHED. One student opted out of the research; 263 students provided informed consent. The number of responses per program and question prompt are detailed in Table 1. A total of 362 responses across eight discussion prompts were recorded. Response length ranged from three concise sentences to several paragraphs. The conversation “threads” in response to an initial post ranged from one to ten replies. The longest threads related to the panel of community members and the panel of families with transgender youth.

Three major themes were constructed from the data, including (1) The Power to Help or Harm, (2) The Responsibility to Provide Health *Care*, and (3) A Posture of Humility: Listen and Learn. Each theme was supported by three to four subthemes. Illustrative quotes were recorded verbatim (Table 2).

**Table 2:** Major Findings from the Qualitative Data Analysis: Themes, Subthemes, and Illustrative Quotes

Theme	Subthemes	Frequency, *n* (%)	Illustrative Quotes
The Power to Help or Harm	Health care providers	117 (32)	“This mom said they had a wonderful experience with their pediatrician, who “never missed a beat” and just allowed [Taylor] to be [Taylor]. While she expressed gratitude about her family’s experience, she did share that [a nearby hospital] will not care for transgender children. This was incredibly disheartening to hear. This highlights the many existing opportunities within healthcare for providers to advocate for their patients and become better allies to the LGBTQ+ community.” “Medicine is a field about human interaction where the type of care we provide and the trust we have with our patients are always fluctuating. I think it is our responsibility to meet our patients where they are and work with our patients to provide the care that everyone deserves. Hopefully by interacting with different people and learning about the stories of others, we will be able to more strongly empathize with the struggles that the patients are going through. That way we do not add to the problem, but can be support that these families can lean on.”
	Family	65 (18)	“One of the TransParent-ing members shared her own experiences transitioning as an adult and how she wished she’d had support from her family at a young age because she was in her fifties by the time she felt that she could be authentically herself. While this was heartbreaking, it was so informative about the emotional toll of not feeling supported by family members.” “I think there is a misconception that parents have to do extravagant things in order to provide support and a comfortable environment for kids to express themselves. While this may be important to some, his mom’s acknowledgment of his gender and openness to expression gave him the space to be authentically himself. She addressed this when she said “I didn’t parent any of my kids differently”. Creating an open and welcoming environment just requires unconditional love and open communication.”
	Faith communities	25 (7)	“Faith communities can benefit the health outcomes of transgender patients by establishing an atmosphere of compassion rather than condemnation so as to avoid traumatizing and hurting these individuals. Something I wish faith communities realized is that unconditional love does not mean unconditional acceptance; you don’t have to agree with how someone lives their life in order to love them. Love is action and if more individuals accepted this, it could have a huge impact and improve the quality of life, and thus overall health, of transgender patients that live in a society where they must constantly worry about harassment, rejection, etc.” “If transgender patients feel they are being persecuted and discriminated against, they will naturally tend to have more anxiety and potentially worse mental health outcomes. On the contrary, faith communities, if they are supportive and caring, can be a great safety net and support system for transgender patients.”
The Responsibility to Provide Health *Care*	Responsibility to be educated	63 (17)	“It did seem like most of the problems that the panel members faced were because of lack of knowledge about the transgender community. Lack of knowledge is not an excuse to give someone a bad experience in healthcare. Medical professionals should be able to treat any person while also recognizing the intersectionality of the people they see.” “I think the most impactful thing I heard was when one of the panel members was repeating stories she heard of people going in for the healthcare appointments where the physicians did not really know or understand their anatomy (ie. not knowing the patient did not have a uterus). It really emphasized how important it is that we be educated on different procedures and hormone therapies that are involved in transitioning because it would be dangerous to treat transgender patients by just making guesses or assumptions”
	Responsibility to build trust	21 (6)	“We have to remember that we may be healthcare professionals, but this is still a partnership and we don’t know more about a person than they do, so in order for us to give them proper information to make decisions about their care, we have to ensure that they feel as if they can even trust us enough to be open about sensitive topics.” “Using the correct language when caring for transgender patients and clients is affirming to them and helps create a trusting relationship between the provider and patient. This relationship of trust is important in the health care setting because it means more information is probably shared with the provider, which leads to more accurate diagnosis and treatment.”
	Responsibility to create a safe space	50 (14)	“The panel members mention how the doctor’s office is a place where they feel uncomfortable and nervous, so whatever I can do to give them a safe environment will be put in place… It is important that members of the transgender community feel comfortable and safe within a hospital/ therapy setting, as it may be the first time.” “I hope that as physicians, and just people in the community, we are able to cultivate a supportive and welcoming space that doesn’t require transgender youth to have to prepare themselves for the outside world by growing that thicker skin.”
	Navigating professional and personal identities	46 (13)	“Even though we are not required to provide gender affirming care, especially if it is contrary to religious beliefs, it is important to be able to understand patients perspectives to refer them to where they can go. Many of these patients experiencing gender dysphoria are undergoing internal conflict, and it is important to refer them to the resources they want if we are unable to or are morally prevented from providing them ourselves.” “Everybody is entitled to their opinion and point of view, and no one is called to affirm or support a view especially if it may require us giving up our own…Judgement is never ours to give, but faith informs many of us of the humanity in each other. Recognizing this humanity is easy and universal, and is a way to support these communities.” “I think this breakout session gave me a lot to think about as an individual who grew up in the Catholic Church and attended Catholic schools throughout my life. One individual during this breakout session asked a question regarding the future for the Catholic Church and if BLINDED FOR PEER REVIEW believes there will be any changes made to support those who are transgender and non-conforming. BLINDED FOR PEER REVIEW believed it is important to start conversations surrounding this topic and the Catholic Church. I think one way we can start this change is through discussion with family members and other members in the Catholic Church. One way I think we can do this is by loving and accepting everyone and their differences. As healthcare providers, we can start these conversations with other healthcare professionals and be allies for those within the community.”
A Posture of Humility: Listen and Learn	Centering transgender narratives	127 (35)	“Learning of the different medical experiences of transgender people was surprising to me. I expected to hear some of that information, but hearing it still surprised me nonetheless. It’s heartbreaking to know that gender diverse people can’t be treated with respect, but it’s also sad that general information about the community is lacking within medical professionals.” “Hearing the panel members discuss how they have been discriminated against in the health field was a very eye opening topic for me as well. I liked how you mentioned that these situations don’t happen for people who aren’t transgender, so they shouldn’t happen for those who are. I think this is an important topic to bring to light because everyone should be treated equally, regardless of how you identify. Transgender individuals should not have to feel uncomfortable or nervous in any environment, especially the doctor’s office. To hear about the disrespect they have been shown in a place where they should feel the most safe pushes me even further to want to create an environment that is inviting and accepting of all people.”
	Openness to continuous learning	27 (7)	“I think it’s great to acknowledge the limits of our knowledge and to be open to learning more so that we can help our future patients. It’s humbling to realize that there are lots of considerations for our future patients that we may not have ever thought about or even realized had to be considered.” “As clinicians, we have an ethical responsibility to provide culturally competent care, which requires an understanding of the array of identities and expressions that transgender and gender-nonconforming people represent. Once we understand what culturally competent transgender care looks like, we must continue to educate ourselves, actively listen to community members, and recognize and address our personal biases.”
	Practical takeaways	73 (20)	“I also realized during the panel how much practice it can take when you are not used to using this language. As future physicians, using inclusive language is key to establishing a productive healthcare provider and patient alliance. We want to respect the patients by using inclusive language so that they can focus on their health, rather than whether their provider will discriminate against them.” “‘Practice makes progress, not perfection’ is one line that stuck with me today. As healthcare professionals there is always room to better your treatment and self to be inclusive to everyone.”

### The power to help or harm

The theme, the Power to Help or Harm, was formed from participant reflections regarding the influence of health care providers, family members, and faith communities on the health and well-being of the transgender community. Narratives of both negative and positive experiences in health care from the transgender community panel informed participants’ discussion of how they, as future health care providers, will be positioned to impact the care their transgender patients receive; this consciousness of power dynamics is an essential element of critical pedagogy [40]. Participants observed how negative experiences will “alienate” or “ostracize patients,” “add stress,” “restrict people,” and “disrupt care,” while positive experiences will “build trust,” “build rapport,” and “respect the dignity” of their patients. This discourse centered primarily on the importance of gender-inclusive language and communication, but also extended to the range of experiences throughout a visit, such as elements of the physical environment (i.e. signage, bathrooms), the design of clinical intake forms, and interactions with other staff. Participants conveyed a sense of hope for the future that “this generation of healthcare providers will be different” and can “role model inclusivity,” while also recognizing that ongoing education is needed or “one day of training isn’t enough.”

Next, participants discussed the power of a patient’s family to help or harm a transgender patient’s health and well-being, which has been well-established in existing research [45–47]. This subtheme emerged from observations of the variability in parental and sibling support described in the panel discussion. For some, the recognition that transgender youth may not feel supported at home underpinned the need to be supportive as a healthcare provider; one participant noted, “It is important for us to create a welcoming and caring environment for that child, especially if they are not receiving that at home.” Discussion also centered on how families, especially parents, need to feel “connected,” “supported” or “have an outlet” to “better support their child at home,” and that healthcare providers can be supportive by referring families to support groups and resources. Similarly, Mucha and colleagues reported that parents of transgender children face challenges such as lack of knowledge and insecurity, and that education for families may improve the home environment [48].

The Power to Help or Harm, or “compassion rather than condemnation,” was heightened throughout the discourse on faith communities. Participants overwhelmingly endorsed the need for faith communities to “accept,” “welcome,” “protect,” “respect” or “love” the transgender community. However, views on the way in which faith communities care for transgender members diverged when tapered down to acceptance of gender diversity, or the assertion that “unconditional love does not mean unconditional acceptance.” One participant noted, “From my own faith community, The Catholic Church, we accept only two genders, male and female…however, persons undergoing the internal conflict between sex and gender should be treated with respect, justice, and charity.” Other participants critiqued this notion as “damaging” in that “Simple tolerance of someone’s existence doesn’t address current inequities.” One participant who self-identified as Muslim critiqued this notation as “damaging”:

The approach you have described fails to acknowledge the material disparities LGBT + people face, and thereby does the community a degree of injustice…Treating everyone with respect and dignity is easy to articulate as a modus operandi, but I think it amounts to “don’t ask, don’t tell” when in response to systemic issues in our society. In other words, I think saying we should treat people with respect and dignity is an invitation to stop deliberating on complex issues when those issues might challenge the beliefs we hold dear.

Participants also reflected on the conflict between their current views and their religious upbringings and were refreshed by the idea that they could practice a religion and still disagree with certain tenets. One participant commented, “…my own personal journey over the last several years made me realize that how I practice my religion (Hinduism) may be different from how others practice it. I don’t believe everything that our religious leader says, and that’s okay.”

### The responsibility to provide health care

While the previous theme captured the influence of the broader healthcare system, the theme, Responsibility to Provide Health *Care*, was primarily shaped by reflections on the roles of individual healthcare providers to be “educated,” “competent,” “prepared” or “equipped” to *care* for a transgender patient above all else. This finding aligned with existing studies confirming students’ appreciation for training on transgender health and the use of evidence-based medicine [11, 19, 31]. The importance of being educated on transgender health, crystallized from narratives shared by the transgender community panel, revealed striking gaps in provider competence. One participant reflected:

I was most surprised when a panel member stated when they…went to a physician’s office to do a checkup and the health-care provider said their cervix was fine. However, it turns out the patient did not have a cervix…This example demonstrates a gap in providers’ knowledge regarding trans/queer bodies, which is scary and impacts their quality of care.

Participants also distinguished that healthcare providers should be educated, but not by their patients “when they are sitting in your office.” One participant commented, “We don’t ask any other population to do that.” Another remarked doing so is “inappropriate as a clinician—we are there to serve them, after all.” However, participants also noted the importance of learning from the transgender community, which is further detailed in the description of the last major theme.

Next, the Responsibility to Provide Health *Care* also encapsulated the responsibility to provide a “safe space” and to “embrace the humanity” of each patient. In other words, providing health *care* for a patient meant not just being knowledgeable, but also facilitating a safe and welcoming space; this aligned with existing research that LGBTQ educational interventions positively impacted student attitudes to be more welcoming or inclusive [34]. These subthemes were constructed from reflections of overt discrimination transgender community panel members had experienced. Participants described their reactions as “shocked,” “surprised” and “eye-opening.” One participant reflected,

The most surprising/revealing part of the panel where members discussed their experiences was hearing about times they were discriminated against. There were stories about how medical procedures were refused during an emergency, misgendering on purpose during a medical visit because the doctor “was not comfortable” or “didn’t believe”, or when the doctor did not know the anatomy of an individual who had received gender-affirming surgeries. We hear about situations like this through social media but hearing it in person shows just how serious these situations can be.

Another participant reacted to a narrative where the provider refused to provide services when learning of the patient’s transgender identity: “This story specifically touched me because I assumed that as a healthcare provider, we are all taught to provide respected [respectful] care to each individual we see. I was deeply saddened to find out that this is not always true.”

Lastly, the discourse revealed a degree of negotiating participants’ own professional and personal identities, or “how a physician’s own belief system impacts care.” [22]. Many participants freely and enthusiastically declared their commitment to be a gender-affirming healthcare provider to the best of their abilities. One participant commented “Hearing about these experiences will influence my future clinical practice because I will go into each patient visit with as much preparedness and knowledge as possible.” Another noted, “In my future practice I will try to go into patient visits without making assumptions on their gender identity or anatomy and seek to create a safe environment for my patients.” Others expressed the need for themselves, or other healthcare providers, to separate their professional and personal identities in order to provide quality care. One participant described, “As medical professionals, we must give the best possible care to every single one of our patients, no matter how we feel about the situation.” Another exclaimed, “I adamantly defend and support the right to keep your own views while being able to simultaneously respect the transgender community, providing effective care, giving respect and latitude to dissenting opinions, and most importantly, not denying anyone’s humanity.” Therefore, although there was strong consensus on the Responsibility to Provide Health Care, the underlying logic or motivation for doing so varied. Similarly, after a sensitivity training program for Kenyan healthcare workers who provided care for men who have sex with men, participants clarified their roles as a professional versus their roles as a citizen (“As a clinician, my duty is to treat without imposing my values on the patient. That’s the positive thing I got from [the training] and it’s what I’m doing now.)” [49].

The concern of secondary stigma, or stigma against healthcare providers for caring for a marginalized group, [50] arose in existing research [49]. However, this was not a finding of the present study. This may be explained by the study population of students who were not yet aware of this possibility, lack of concern for this type of stigma, or that the discussion questions did not prompt them to consider this possibility.

### A posture of humility: listen and learn

The final theme, A Posture of Humility: Listen and Learn, was constructed from participant reflections on the importance of learning directly from the transgender community and staying open to continuous learning. Participants described the need to know “what is most important to my patients,” “to meet patients where they are at,” “to understand their lived experiences” or “to acknowledge the subjective and individual experiences our patients feel.” This approach was described as forming a “patient alliance” or “joint effort” with a patient. One participant reflected:

As an outsider to this community, I can educate myself as best as possible, but I will never truly understand the struggles and hardships each of them has experienced throughout their journey. However, as a healthcare provider, it is my role to listen and learn from these individuals, so I can provide them with a positive interaction.

Although participants recognized a responsibility to be educated on the basics of transgender health, they also expressed humility in that “There is so much more to learn and one day of training isn’t enough.” Another suggested that “progress not perfection” should be the goal. A third participant noted, “I agree that making a sincere effort to be inclusive instead of aiming for perfection is a good and more realistic place to start.”

Participants also expressed hope that others would receive similar training on transgender health, especially first-person accounts from transgender patients. One participant commented, “I would also hope that other future or current medical providers will have the opportunity to hear these sorts of stories and perspectives as motivation to adopt this more inclusive language.” Another participant expressed, “Although there is a lot of room to make improvements on and add additional education on this topic in medical school, we can also stress the importance of this topic to future medical students. We will have many opportunities to share both what we learned today and what we will continue to learn over time with other health care professionals and students in training.” This finding mirrored existing students where medical students expressed the importance and relevance of this topic to their medical education [19].

Lastly, participants shared numerous practical takeaways to demonstrate what they had learned from the education, such as: limiting clinical questioning to relevant information; informing patients why certain questions are being asked; ensuring intake forms are gender-inclusive; refraining from making assumptions about a person’s gender identity; using open-ended questions; and creating an open and welcoming physical environment. Though evidence supports that educational interventions can effectively result in a short-term improvement in knowledge, scholars have underpinned the need to explore whether knowledge is retained long-term [12, 13, 28].

### Considerations for clinical education

Whereas existing pre-test, post-test survey research supports that transgender health education can significantly impact student attitudes towards the transgender population, [7, 10, 15, 25, 27, 35] the findings of this study offer clinical educators a rich, in-depth description of student perceptions of transgender health education. Clinical educators may face barriers when implementing transgender health education content into their curricula, including pushback from students who have received different messages about gender diversity from their families, religious leaders, political leaders, or society at large [47]. Thus, a deep understanding of student perceptions can help clinical educators better understand their students’ viewpoints and harness what drives them to learn. For example, the subthemes, Responsibility to be Educated, Responsibility to Build Trust, and Responsibility to Create a Safe Space revealed students’ expectations for how all healthcare providers should deliver healthcare. These expectations were closely aligned with the Jesuit value of Cura Personalis, or care for the whole person. Drawing on these expectations may help students find common ground, even though their views on gender diversity may diverge.

The study findings may be particularly useful to religiously affiliated institutions. To our knowledge, this is the first study to evaluate transgender health education at a Catholic, Jesuit institution. We welcomed discourse on religion and transgender health in multiple ways, including a welcoming address to the ITHED from a Catholic religious leader, a breakout discussion with the religious leader and her experiences of ministering within the transgender community, and a discussion prompt inviting students to reflect on these sessions. The resulting theme The Power to Help or Harm and the subtheme “Faith Communities” provided a rich description of how students view the impact of faith communities on transgender individuals, with a notable intolerance for the harms caused. Critical pedagogy offers an opportunity for students to reflect on harmful societal norms. Especially when confronted with narratives of mistreatment at the hands of healthcare providers, students may be moved to critique and challenge the societal conditions that allowed for these cases to occur, which is at the heart of critical pedagogy [40]. One participant commented:

I thoroughly enjoyed what Nancy had to say in this breakout session. I think that often times we associate peoples’ lack of acceptance with age and or religion, but I believe that she cleared up the fact that there should literally be no correlation between those things, especially no correlation between identifying as a Christian/Catholic and being homophobic/transphobic (and more).

Relatedly, the theme The Responsibility to Provide Health *Care* and the subtheme “Navigating Professional and Personal Identities” captured how students considered the intersection of their personal values, which often included religion, with their professional identities. Clinical educators may be encouraged to embrace discourse on how different religious identities, or the missions of religiously affiliated institutions, intersect with transgender health. One participant reflected:

I was also raised Catholic and grew up in Catholic schooling my entire life. I was curious what Nancy would have to say about those who are transgender and non-conforming from a Catholic perspective, and her take on the topic gave me hope. She said that no matter who they are, every person should be accepted, respected, and loved…I imagine myself talking about the topics we discussed at ITHED with my grandparents and older relatives, and I predict these conversations to be frustrating. We have been conditioned to live in a binary society in which there is male and female, and it can be confusing to people who have held this to be true for so long. However, it becomes a problem when someone takes their confusion and portrays it as hatred, un-acceptance, or disrespect. Ideally, these conversations can be met with questions, curiosity, and an openness to learn rather than a closed-off mindset.

Next, the ITHED consisted of lectures provided by transgender educators, panel discussions, a guided reflection, and breakout sessions. While the lectures provided foundational knowledge in transgender health and communication, students described the panel discussions (one with transgender community members, and a second with the parents of transgender youth) as the most “memorable” and “powerful” sessions of the day. Facilitated discussion with individuals who could share their lived experiences of being transgender were particularly impactful on students’ experiences. Similarly, Park and Safer reported higher levels of knowledge and comfort in caring for transgender patients through clinical exposure, where students had genuine, face-to-face conversations with transgender patients, compared to the effect of didactic content alone [18]. Findings of a systematic review indicated that most educational interventions have not been led by members of the LGBTQ community; [34] thus, clinical educators are encouraged to integrate pedagogical approaches that feature first person accounts, such as panel discussions, videos, or simulation [51–55] Partnering with community-based organizations can ensure the approach is authentic, non-stigmatizing, and protects the emotional well-being of the panelists when sharing difficult and often traumatic experiences [17, 31, 44].

The research team also observed student comments regarding the role of one’s religion or faith community in caring for transgender patients, even when viewpoints starkly diverged. One participant commented that this discussion in a breakout session helped “…bridge the subjective disconnect between faith and transgender health.” Another participant commented, “As both a religious individual and someone that trusts science, I’m very happy to hear this topic being discussed.” Despite some disagreement, the discourse for this breakout session was respectful, constructive, and thought-provoking. After a robust conversation, one participant stated, “This is what higher education is supposed to be about, and thank you, for your thought provoking reply.” Therefore, clinical educators are encouraged to facilitate opportunities for students to explore the intersection of their personal and professional identities in a healthy and productive manner, which is an essential element of both critical pedagogy and the Jesuit practice of reflection [40, 42].

Lastly, the research team observed that the ITHED held special meaning for students who self-identified as transgender or as part of the broader LGBTQ + community. One participant commented, “I look forward to proudly wearing my pride symbol on the wards of this Jesuit institution.” Another student shared with his program director that he had never felt so safe on-campus. Thus, clinical educators may consider how their own LGBTQ + students may be feeling during an education of this nature, and how to best care for their well-being.

### Strengths, limitations, and future research

This study was strengthened by triangulation of the data across four medical and allied health disciplines, the interdisciplinary nature of the research team and study participants, and use of a reputable quality assurance checklist appropriate for the study design 43]. A limitation of the study was reliance on written student reflections and subsequent discussion; although the assignment description was preceded by an informed consent statement, students may have been hesitant to opt out of having their submissions used for research purposes. Responses may have been impacted by social desirability bias given that they were required to respond to their peers 55]. Furthermore, students may have responded to more general questions in ways that reflected their existing views (i.e. How have societal messages about gender evolved over the past several decades?) rather than resulting from participation in the ITHED; future research could employ a mixed methods strategy to better quantify changes in perceptions due to transgender health education.

Extrapolation of the study findings, or the application to other settings, was limited by data collection at a single Midwestern, Catholic, Jesuit institution; future research can examine student perception of transgender health education at institutions in other regions throughout the United States and with other religious affiliations where the climate surrounding transgender health may differ. Given the breadth of pedagogical approaches utilized in the existing research, future studies may also examine student perceptions of videos, simulations, and clinical rotations centered on transgender health. Lastly, given that the researchers did not compare the responses of one discipline to another, future research may explore differences in how transgender health education is perceived across disciplines.

## Conclusion

Health professional students were highly receptive towards transgender health education delivered by transgender community members. First-person accounts of both positive and negative experiences in healthcare are particularly effective at illustrating the power of healthcare providers to help or harm transgender patients. Reflection and constructive dialogue offered students an opportunity to explore their identities as healthcare providers at the intersection of their religious and cultural beliefs.

### Electronic supplementary material

Below is the link to the electronic supplementary material.

Supplementary Material 1
