# Supplementary material for: The power to help or harm: student perceptions of transgender health education using a qualitative approach
Source: BMC Med Educ. 2023 Nov 7;23:836. doi: 10.1186/s12909-023-04761-9 (PMC10629163; doi:10.1186/s12909-023-04761-9)

2022 INTERPROFESSIONAL TRANSGENDER HEALTH EDUCATION DAY (ITHED)

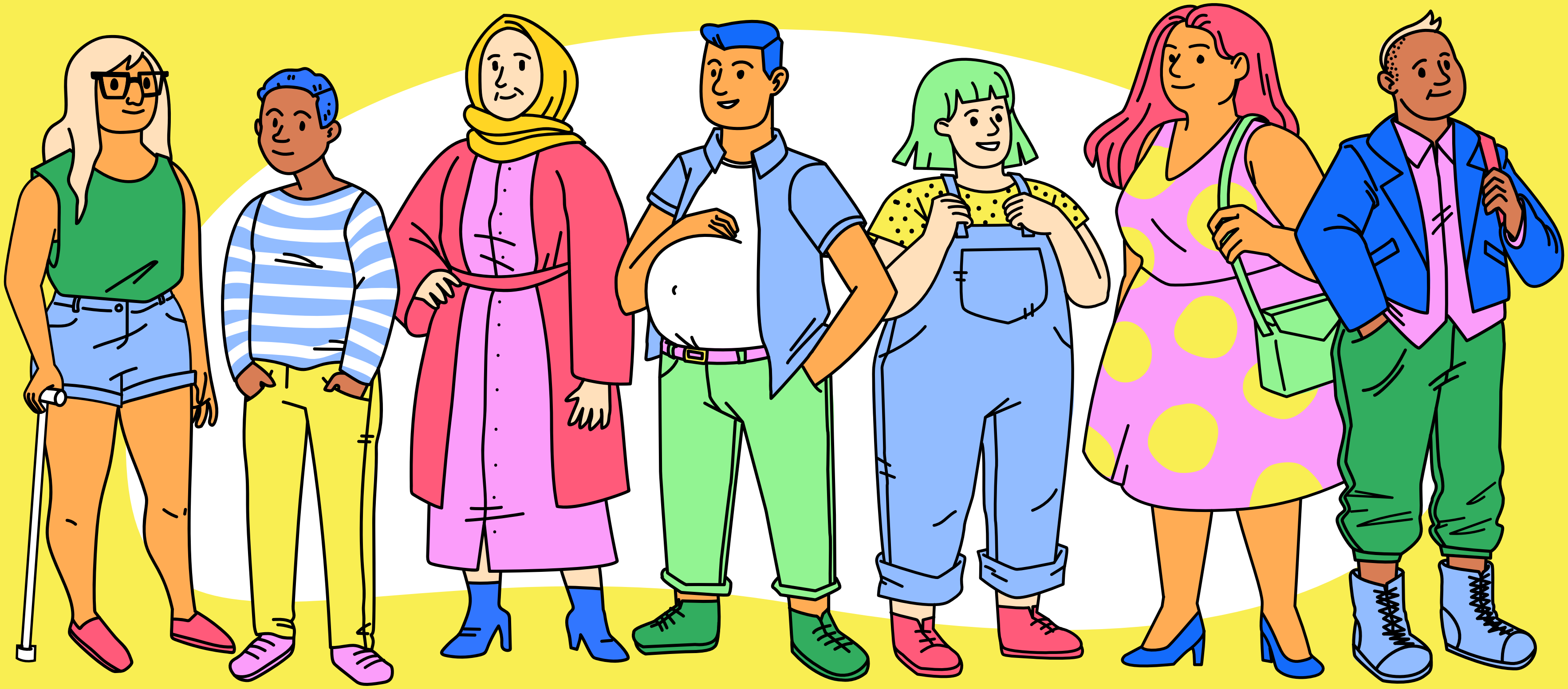

SAINT LOUIS UNIVERSITY™

INTERPROFESSIONAL TRANSGENDER HEALTH EDUCATION DAY | 2022

CURRICULUM

Intended Audience

College/university students enrolled in clinical education or training programs, current healthcare professionals and preceptors.

Description

The ITHED is designed to provide foundational knowledge on transgender health and gender-inclusive communication students and current healthcare professionals.

Evaluation will involve participation in online discussion forums specific to the main sessions and breakout session topics.

Materials

Suggested resources include:

- The World Professional Association for Transgender Health (WPATH) Standards of Care for the Health of Transsexual, Transgender, and Gender Nonconforming People, Version 8
- University of California San Francisco Guidelines for the Primary and Gender-Affirming Care of Transgender and Gender Nonbinary People, 2nd edition
- The Report of the 2015 U.S. Transgender Survey by the National Center for Transgender Equality
- Endocrine Society Clinical Practice Guidelines

Methods of Instruction

The ITHED methods of instruction include interactive lectures, guided reflection, a panel discussion, and interactive breakout sessions.

Learning Objectives

After participating in the ITHED, students will be able to:

1. Identify prominent physical and mental health disparities that impact the transgender population.
2. Discuss the challenges transgender patients face within the healthcare system.
3. Describe aspects of gender-inclusive language and communication.
4. Articulate strategies to promote a gender-inclusive clinical environment.
5. Locate reputable resources to inform clinical practice within one's profession.
6. Identify the role of various health professionals in caring for a transgender patient.

Description of Learning Activities

- ***Trans 101:*** Introduces fundamental concepts in transgender health, health disparities that impact the transgender population, and gender-inclusive language and communication (learning objectives 1-3).
- ***Trans 102:*** Addresses forms of transitioning, including social, legal, and medical transitioning (learning objectives 3-4).
- ***Trans 103:*** Provides discipline-specific considerations and details the role of various healthcare providers in the care of a transgender patient (learning objectives 5-6).
- ***Guided Reflection on Gender Identity:*** Invites students to reflect on their own gender identity and its context in society.
- ***Panel of Transgender Community Members:*** Provides space for transgender and gender diverse community members to share about their experiences in healthcare (learning objective 2).
- ***Dynamic Breakout Sessions:*** Allows students to explore greater depth of understanding on a variety of topics in small groups (learning objectives 1-6).

INTERPROFESSIONAL TRANSGENDER HEALTH EDUCATION DAY

OCTOBER 14, 2022 / SCHEDULE

Time	Content	Presenter(s)	Location
8:30 AM - 8:45 AM	Welcome	Nancy Corcoran	LRC ABC
8:45 AM - 9:30 AM	Trans 101: Introduction to Transgender Health	Beth Gombos	LRC ABC
9:30 AM - 9:45 AM	Guided Reflection on Gender Identity	Katie Heiden-Rootes	LRC ABC
9:45 AM - 10:00 AM	Break		
10:00 AM - 11:00 AM	Trans 102: Introduction to Gender-Affirming Medical Care and Transitioning	Beth Gombos, Theresa Drallmeier	LRC ABC
11:00 AM - 11:15 AM	Break		
11:15 AM - 12:15 PM	Panel Discussion with Transgender Community Members		LRC ABC
12:15 PM - 1:00 PM	Lunch		
1:00 PM - 1:45 PM	Trans 103: Discipline-Specific		
	• Medical & PA Students	Theresa Drallmeier	LRC ABC
	• Medical Family Therapy Students	Katie Heiden-Rootes	LRC 111
	• Speech, Language and Hearing Sciences Students	Emily Buxbaum	LRC 112-113
	• Nutrition and Dietetics Students	Whitney Linsenmeyer	LRC 110
	• Occupational Therapy Students	Wendy Stav	LRC 108
2:00 PM - 2:45 PM	Dynamic Breakout Sessions, Round 1 (see next page for session descriptions)		
	• Follow-Up Discussion with Panel Members	Beth Gombos	LRC A
	• Sex Under the Gender Expansive Umbrella	Willow Rosen	LRC B
	• TransParent-ing: A Panel Discussion with Families of Transgender Youth	TransParent, Jae Seevers, Quin Rich	LRC C
	• Gender-affirming Language and Communication: A Skills Workshop	Rabia Rahman, Emily Buxbaum	LRC 111
	• Loitering with Intent: A Conversation with Nancy Corcoran, CSJ	Nancy Corcoran	LRC 110
	• PrEP and HIV in the Gender Diverse Population	Michael Donovan	LRC 112-113
	• "But What About..." Safe Space to Ask Lingering Questions	Patrick Cousins	LRC 108
	• Restorative Space for Transgender and Gender Diverse Students		LRC 105-106
3:00 PM - 3:45 PM	Dynamic Breakout Sessions, Round 2 (see next page for session descriptions)		
	• Follow-Up Discussion with Panel Members	Beth Gombos	LRC A
	• Sex Under the Gender Expansive Umbrella	Willow Rosen	LRC B
	• TransParent-ing: A Panel Discussion with Families of Transgender Youth	TransParent, Jae Seevers, Quin Rich	LRC C
	• Gender-affirming Language and Communication: A Skills Workshop	Rabia Rahman, Emily Buxbaum	LRC 111
	• Loitering with Intent: A Conversation with Nancy Corcoran, CSJ	Nancy Corcoran	LRC 110
	• PrEP and HIV in the Gender Diverse Population	Michael Donovan	LRC 112-113
	• "But What About..." Safe Space to Ask Lingering Questions	Patrick Cousins	LRC 108
	• Restorative Space for Transgender and Gender Diverse Students		LRC 105-106
3:50 PM - 4:00 PM	Closing Remarks	Beth Gombos	LRC ABC

INTERPROFESSIONAL TRANSGENDER HEALTH EDUCATION DAY | 2022

BREAK-OUT SESSION DESCRIPTIONS

Follow-up Discussion with Panel Members

Following the "Panel of Transgender Community Members" earlier in the day, this will be an opportunity to continue the dialogue with transgender individuals and their experiences in healthcare.

Restorative Space for Transgender and Gender Diverse Students

This restorative space is reserved for students who identify as transgender or gender diverse. This will simply be a quiet opportunity to rest; no structured programming will be delivered.
***Advance sign-up is not required! Please utilize the quiet space as needed.

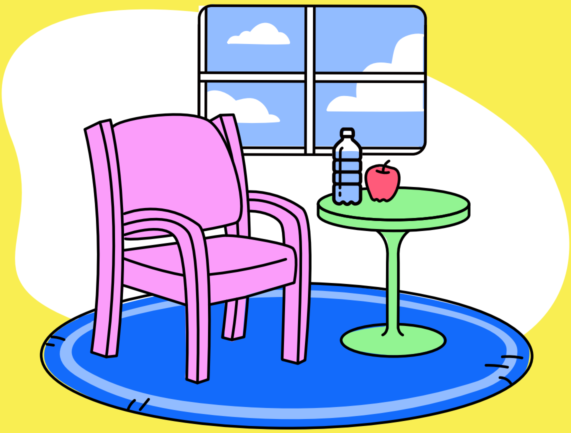

PrEP and HIV in the Gender Diverse Population

This session addresses the basics of pre-exposure prophylaxis (medication designed to reduce HIV contraction from sex or IV drug use) and HIV management, with a focus on the gender diverse population. This session will be led by Michael Donovan, MD, a family physician at Southampton Healthcare who provides primary care and HIV management for the LBGTQ community.

Gender-Affirming Language and Communication: A Skills Workshop

This interactive workshop will provide students with the opportunity to practice gender-affirming language and communication, including use of names and pronouns, gender-neutral terminology, and how to handle communication mistakes. This session will be led by Emily Buxbaum, MS, CCC-SLP who provides voice therapy to gender diverse folks through the Speech-Language and Hearing Clinic, and Rabia Rahman, PhD, RD, LD, who publishes on the role of registered dietitians in the care of transgender clients.

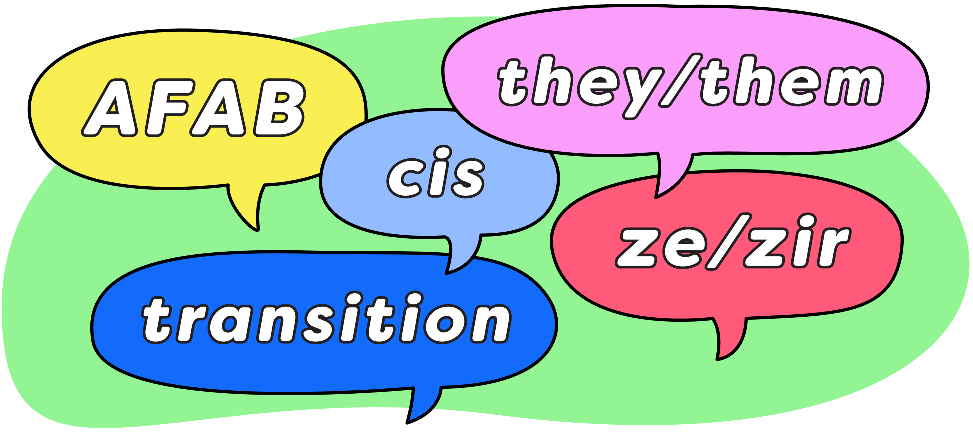

BREAK-OUT SESSION DESCRIPTIONS

"But What About..." Safe Space to Ask Lingering Questions

This session will be an open dialogue led by SLU campus minister Patrick Cousins with students who have lingering questions about biological sex, gender identity, society, and/or the role of religion. Students can bring questions that may have arisen from their personal upbringing, religious practice, or from the media.

Sex Under the Gender Expansive Umbrella

A short session touching on some of the realities of sexual and reproductive health for gender expansive and transgender people. This will take into consideration some factors such as Gender Affirming Hormone Therapy (GAHT) / Hormone Replacement Therapy (HRT), surgery and adaptive wear. This session will be led by Mx. Willow Rosen a sexual and reproductive health care educator with over a decade of experience helping transgender and queer individuals navigate this ever expanding landscape of care.

Loitering with Intent: Conversation with Sister Nancy Corcoran

Nancy Corcoran, CSJ defines her ministry as one of presence and accompaniment with the LGBTQIA population. She notes, "We [the Sisters of Saint Joseph of Carondelet] recognized the ignorance and intolerance surrounding humans who claim membership in the LGBTQIA communities. We promised to stand in solidarity and support, especially these folks in our quest for inclusion and justice, knowing that Jesus welcomed everyone to the table, respecting the dignity of every human person." For more information on her work, visit: https://www.csjsl.org/news/a-ministry-of-accompaniment

TransParent-ing: A Panel Discussion with Families of Transgender Youth

TransParent is a national organization with local chapters throughout the country. Their mission is to bring compassionate support to parents and caregivers navigating complex issues faced by gender-expansive individuals. In this panel discussion, parents will discuss their family's journey, the experiences they've had in the healthcare system, and their hopes for the future of healthcare. The panel will be moderated by Jae Seevers, a student therapist the Medical Family Therapy Program, that works with trans youth and their families. Questions from the audience are encouraged. The panel will be moderated by Jae Seevers and Quin Rich, student therapists the Medical Family Therapy Program who work with trans youth and their families.

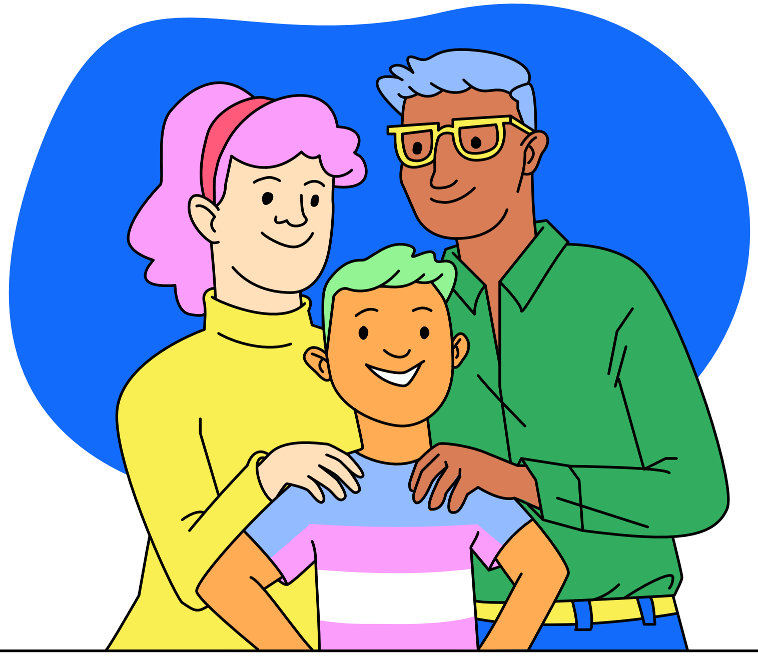

Supplement: Supplementary file 1 — Supplementary Material 1 [file 12909_2023_4761_MOESM1_ESM.pdf]
